# The role of long-term mechanical circulatory support in patients with advanced heart failure

**DOI:** 10.1007/s12471-020-01449-3

**Published:** 2020-08-11

**Authors:** S. E. A. Felix, N. de Jonge, K. Caliskan, O. Birim, K. Damman, M. Kuijpers, L. F. Tops, M. Palmen, F. Z. Ramjankhan

**Affiliations:** 1 Department of Cardiology, University Medical Center Utrecht, University of Utrecht, Utrecht, The Netherlands; 2 Department of Cardiology, Erasmus MC University Medical Center, Rotterdam, The Netherlands; 3 Department of Cardiothoracic Surgery, Erasmus MC University Medical Center, Rotterdam, The Netherlands; 4 Department of Cardiology, University Medical Center Groningen, University of Groningen, Groningen, The Netherlands; 5 Department of Cardiothoracic Surgery, University Medical Center Groningen, University of Groningen, Groningen, The Netherlands; 6 Department of Cardiology, Leiden University Medical Center, Leiden, The Netherlands; 7 Department of Cardiothoracic Surgery, Leiden University Medical Center, Leiden, The Netherlands; 8 Department of Cardiothoracic Surgery, University Medical Center of Utrecht, University of Utrecht, Utrecht, The Netherlands

**Keywords:** Left ventricular assist device, Advanced heart failure, Survival, Adverse events

## Abstract

In patients with end-stage heart failure, advanced therapies such as heart transplantation and long-term mechanical circulatory support (MCS) with a left ventricular assist device (LVAD) have to be considered. LVADs can be implanted as a bridge to transplantation or as an alternative to heart transplantation: destination therapy. In the Netherlands, long-term LVAD therapy is gaining importance as a result of increased prevalence of heart failure together with a low number of heart transplantations due to shortage of donor hearts. As a result, the difference between bridge to transplantation and destination therapy is becoming more artificial since, at present, most patients initially implanted as bridge to transplantation end up receiving extended LVAD therapy. Following LVAD implantation, survival after 1, 2 and 3 years is 83%, 76% and 70%, respectively. Quality of life improves substantially despite important adverse events such as device-related infection, stroke, major bleeding and right heart failure. Early referral of potential candidates for long-term MCS is of utmost importance and positively influences outcome. In this review, an overview of the indications, contraindications, patient selection, clinical outcome and optimal time of referral for long-term MCS is given.

## Dutch contribution to the field

- Since the introduction of continuous-flow LVADs in the Netherlands in 2006, the number of implantations has increased substantially, outnumbering heart transplantation as treatment for advanced heart failure.
- The results after LVAD implantation justify the use as an alternative to heart transplantation.
- Currently, the four implanting centres (UMCU, EMC, UMCG and LUMC) have sufficient capacity for the LVAD implantations needed.
- Early referral to an LVAD-implanting centre is mandatory for optimal timing and outcome of LVAD implantation.

## Introduction

Patients suffering from advanced heart failure despite individualised optimal medical treatment, with or without cardiac resynchronisation therapy, should be considered for heart transplantation or long-term mechanical circulatory support (MCS) [1]. Currently, heart transplantation is still considered to be the gold standard, showing a relatively good median survival of 15 years [2–5]. Meanwhile, long-term MCS is becoming more and more important due to the growing number of heart failure patients together with the decline in the number of donor hearts. First generation left ventricular assist devices (LVADs) were big pulsatile devices with limited durability. Already in 1993, LVADs were used as bridge to transplantation in the University Medical Center Utrecht [6]. From 2006, smaller and more reliable continuous flow devices became available. The short-term outcome was very promising with a 2-year survival of 76% [7]. Since that time, MCS has become an important part of therapy in advanced heart failure and the number of centres in the Netherlands implanting LVADs has increased to four. Outcome parameters are registered per centre and reported yearly to a central European database (EUROpean registry for patients with Mechanical Assisted Circulatory Support, EUROMACS).

LVADs can be used as bridge to transplantation, or as an alternative to heart transplantation, which is known as destination therapy and in some patients as a bridge to decision in case of temporary contraindications. The present situation in the Netherlands is that most patients with an LVAD as bridge to transplantation will have to wait several years before a donor heart becomes available and many patients will never be transplanted at all. In that way the difference between bridge to transplantation and destination therapy is becoming more and more artificial.

**Fig. 1:**
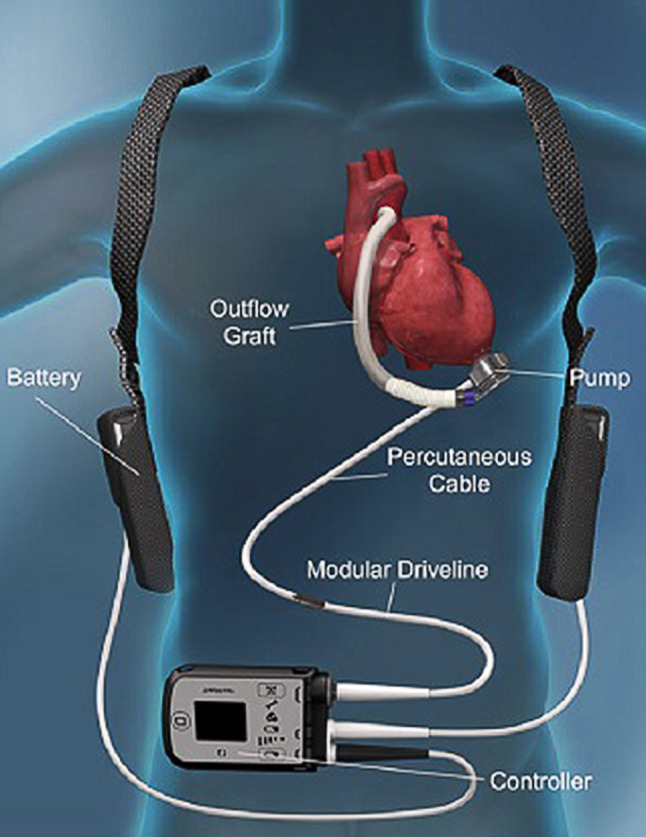
Left ventricular assist device (LVAD)

Currently, the HeartWare Ventricular Assist Device (HVAD) (Medtronic, Framingham, MA, USA) and the Heartmate 3 (HM3, Abbott, St. Paul, MN, USA) are the most frequently used devices for long-term MCS (Fig. 1). The HM3 replaced the Heartmate II (HM-II, Abbott, St. Paul, MN, USA) some years ago, resulting in less need for pump replacements and improved survival free of disabling stroke or reoperation for malfunction than its predecessor [8]. Both HVAD and HM3 are small centrifugal pumps implanted in the pericardial cavity showing very low rates of haemolysis, but necessitating intensive anticoagulation. The percutaneous abdominal driveline is still one of the shortcomings in the design, potentially leading to recurrent or persistent infections.

## Indications for long-term MCS

Indications for long-term MCS generally follow those of heart transplantation. In case of contraindications for heart transplantation, MCS may be considered as an alternative to transplantation in selected patients for which all the below-mentioned criteria also apply:

- Advanced heart failure with a low left ventricular ejection fraction <30% despite optimal therapy consisting of maximally tolerable medication with or without resynchronisation therapy and other interventions to optimise the cardiac condition, as indicated by the current heart failure guideline [2];
- Exercise tolerance, assessed by cardiopulmonary exercise testing, reveals a peak VO_2_ <12 ml/min/kg (<14 ml/kg/min if intolerant to beta blocker) or <50% of the predicted value for age and sex in ambulatory patients: strong intrinsic motivation and Inter-agency Registry for Mechanically Assisted Circulatory Support (INTERMACS) profile 2–6 (Tab. 1; [12])

**Table 1:** INTERMACS classification

NYHA class	INTERMACS profile	Popular term	BTT/DT	Prognosis
IV	1. Critical cardiogenic shock	‘Crash and burn’	NO *	Hours to weeks
IV	2. Progressive decline	‘Sliding fast’	YES	
IV	3. Stable but inotrope dependent	‘Stable dependent’	YES	Weeks to months
IV	4. Recurrent advanced heart failure	‘Frequent flyer’	YES	
IIIb–IV	5. Exertion intolerant	‘Housebound’	To be considered	Months to years
IIIb	6. Exertion limited	‘Walking wounded’	To be considered	
III	7. Advanced NYHA III	NYHA class III	In the long term	

*BTT* bridge to transplantation, *DT* destination therapy, *NYHA* New York Heart Association

## Contraindications for long-term MCS

Patients in cardiogenic shock (INTERMACS profile 1) despite an intra-aortic balloon pump, temporary MCS and/or inotropic support are not eligible for long-term MCS. In addition, a life expectancy of less than 2 years, based on extracardiac disease, is a contraindication for long-term MCS. Furthermore, severe comorbidities may be temporary or persistent contraindications (Tab. 2).

**Table 2:** Contraindications for long-term LVAD therapy

1	INTERMACS 1 (Cardiogenic shock) despite IABP, temporary MCS and/or inotropics
2	Life expectancy <2 years due to extracardiac disease
3	Severe comorbidity/end organ failure – Severe renal failure (estimated GFR <30 ml/min/1.73 m^2^), unlikely to improve after LVAD implantation – Severe liver failure/cirrhosis or portal hypertension, unlikely to improve after LVAD implantation – Severe pulmonary disease (with a FEV1 <1 liter), or pulmonary disease resulting in an important component of symptomatology that could result in de absence of improvement of symptoms after LVAD implantation – Severe central/peripheral artery disease and/or abdominal aorta >5 cm (untreated) – Symptomatic cerebral pathology in the recent 6 months and/or severe disability after neurological event and/or carotid artery stenosis >80% that cannot be treated – Severe neuromuscular pathology, limiting exercise capacity and/or ventilation postoperatively – Increased bleeding risk (which will not improve after LVAD implantation) a. Persisting thrombocytopenia (<50,000 × 10^9^/l) b. Active bleeding c. Severe coagulopathy otherwise – Cognitive or psychosocial factors a. (Beginning) dementia b. Depression, unlikely to improve after LVAD implantation
4	Severe right heart failure, with a high risk for the need for right ventricular assist device (despite in BTT, implanting a biventricular assist device may be considered)
5	Phenotype of heart failure, in which implantation of a LVAD is impossible/complex: – Hypertrophic cardiomyopathy (unless, in dilating phase) – Restrictive cardiomyopathy/endomyocardial fibrosis – Complex uncorrected congenital heart disease/valvular disease
6	Difficulties in ventilation in intubated patients
7	Severe cachexia (BMI <18.5 kg/m^2^), unlikely to be corrected
8	Morbid obesity (BMI >35 kg/m^2^), uncorrected
9	(Increased risk for) systemic infection
10	Severely calcified ascending aorta (where outflow cannula is inserted; consider inserting the outflow cannula at another location)
11	Intolerance to coumarin derivates and/or thrombocyte aggregation inhibitors
12	Non-compliance, substance abuse (drugs/alcohol/nicotin)
13	Absence of social network, severe language barrier

*LVAD* left ventricular assist device, *IABP* intra-aortic balloon pump, *MCS* mechanical circulatory support, *GFR* glomerular filtration rate, *BTT* bridge to translation, *BMI* body mass index

## Patient selection

Patient selection is of utmost importance for outcome after LVAD implantation and is performed by a specialised, multidisciplinary team in LVAD-implanting centres, who take the above-mentioned indications and contraindications into consideration [9].

As mentioned previously, patients in INTERMACS profile 1 (refractory cardiogenic shock) are generally not candidates for long-term MCS directly, but require stabilisation on temporary MCS first, to see if organ function recovers. Primary LVAD implantation in these patients has a proven worse outcome in comparison with patients in INTERMACS profile 2–4 [10, 11].

Besides INTERMACS classification, the evaluation of right ventricular function is very important as there are no reliable options for long-term right ventricular support and right heart failure (RHF) is one of the main complications after LVAD implantation. It is thought to occur in 20–30% of patients, especially early postoperatively after LVAD implantation and is the primary cause of death in 10% [8, 10, 11]. Many criteria are formulated to try to predict perioperative RHF after LVAD implantation. No single criterion suffices, but recently a risk score based on the EUROMACS data was developed, in which invasive pressure measurements, echocardiographic and clinical parameters were combined [12]. Based on this score, a reasonable prediction of early postoperative RHF can be made (C-index of 0.70).

The final decision on LVAD implantation is made by the MCS team (consisting of at least a cardiologist, cardiothoracic surgeon and specialised nurses and technicians) weighing indication, contraindications, right ventricular function, age, previous operations and the ability and willingness of the patient to comply to a complex medical regime against a prospect of potential improvement after LVAD implantation.

With respect to contraindications, potential reversibility has to be analysed, especially with regard to renal insufficiency and hepatic failure [13–15]. Age has to be judged as a biological component in the decision to implant an LVAD. Although there is no absolute upper limit, given the poorer results in elderly people, it is generally not advisable to proceed in patients older than 75 years [1].

## Survival

**Fig. 2:**
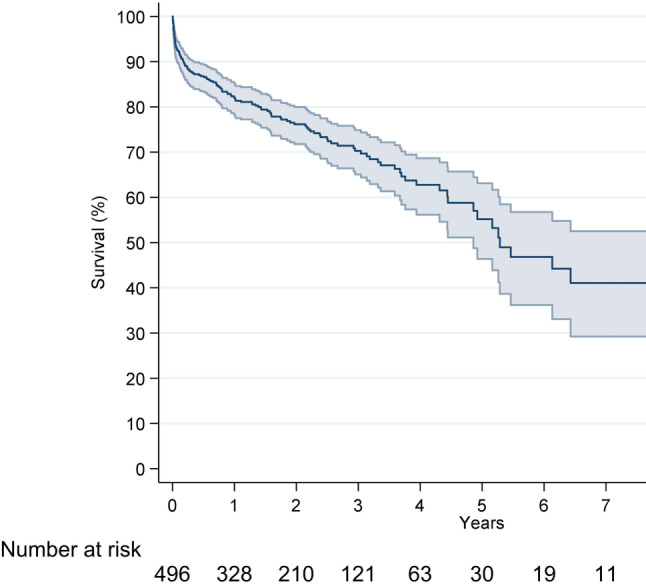
Kaplan-Meier survival curve of patients with an LVAD in the Netherlands, implanted between 2006 and 2019

EUROMACS data, including 2113 patients, demonstrated a survival of 69% (CI 66–71%), 55% (CI 52–58%) and 44% (CI 40–47%) at 1, 2 and 3 years after continuous-flow LVAD implantation, respectively [16]. In the Netherlands, 496 patients (72% male, median age 55 (range 16–74) years) received MCS between 2006 and 2019. Current survival of the four LVAD centres combined is 83%, 76% and 70% after 1, 2 and 3 years, respectively (Fig. 2), with heart transplantation (26%), death (28%), explantation of LVAD (2%) and alive on LVAD on 31 December 2018 (44%) as the endpoint. These data are quite promising given the poor prognosis of the patients before LVAD implantation [17]. Following LVAD implantation, not only survival, but also quality of life and exercise capacity improves impressively, allowing a return to a normal life, including sports activities and even resumption of work [18–20]. Despite this promising survival, morbidity after LVAD implantation remains substantial, as was confirmed in a recent publication showing major bleeding and ventricular tachycardia as the most commonly encountered adverse events [21].

## Adverse events

### Infection

Device-related infections might be limited to the exit site of the driveline but may also extend to other parts of the system. Incidence rates are highest in the first 3 months postoperatively, namely 0.25 events per patient-year. Thereafter, incidence is 0.17 events per patient-year [10]. Patients often require longstanding antibiotic and/or surgical treatment. A recent study identified that the risk for LVAD-associated infections is increased in HM-II when compared with HVAD and in patients who need post-LVAD ICD-related procedures [22]. In the MOMENTUM 3 trial, comparing outcome in HM3 versus HM-II, device-related infections occurred equally in HM3 and HM-II [8].

### Right heart failure

RHF is defined by INTERMACS as increased central venous pressure (>15 mm Hg) with echocardiographic (right heart dysfunction, dilatation and/or significant tricuspid regurgitation) and clinical signs of venous congestion [11]. This may require an increased dose of diuretics and/or inotropics and/or nitric oxide ventilation and/or temporary mechanical support. RHF can occur in the early postoperative phase, but may also develop later in the course of the disease. Patients with late RHF have a worse prognosis in terms of survival and functional capacity, and are more frequently readmitted in comparison with patients without late RHF [23].

### Device malfunction

Device malfunction, including pump thrombosis and driveline-related problems, were most often seen in the HM-II resulting in the need for LVAD replacement. Technical improvement led to almost elimination of pump thrombosis in HM3, as shown in the MOMENTUM 3 trial [8]. However, in HVAD patients, pump thrombosis is still an important problem [24]. In HM3, rare cases of outflow graft twisting have been reported, resulting in decreased pump flow and the need for reparative treatment [25].

### Bleeding

Major bleeding is defined as a suspected internal or external bleeding, resulting in death, rethoracotomy, hospitalisation and/or transfusion of red blood cells (within the first 7 days after the implantation requiring transfusion ≥4 units of packed red blood cells, or any transfusion beyond 7 days postoperatively) [11].

Bleeding is related to the use of anticoagulation and antiplatelet therapy in combination with acquired Von Willebrand syndrome after LVAD implantation as a result of decreased pulsatility [26–28]. This may result in recurrent episodes of gastrointestinal bleeding and nose bleeds.

### Stroke

Patients on MCS may suffer from ischaemic and/or haemorrhagic stroke. In the MOMENTUM 3 trial strokes occurred equally (0.10 and 0.26 events per patient-year, *p* = 0.09, respectively) in both devices during short-term follow-up (31–180 days postoperatively), but beyond this period strokes were 3.3 times less frequently seen with HM3 [28]. Stroke is not only an important cause of morbidity, but also a predictor of mortality [29, 30]. In case of ischaemic or haemorrhagic stroke, the anticoagulation regimen often needs to be revised, thereby increasing the risk for either a haemorrhagic transformation of the ischaemic stroke or pump thrombosis, respectively. This delicate balance between thrombosis and bleeding, known as haemocompatibility, remains one of the major challenges in MCS management.

### Arrhythmias

Ventricular arrhythmias are highly prevalent during MCS (30%), both in the early postoperative phase and later in the course of the disease [31]. Ventricular arrhythmias might be tolerated relatively well (i.e. no loss of consciousness) because output is preserved by the LVAD. However, clinically patients may present with RHF. Most often, the underlying cardiomyopathy leads to ventricular arrhythmias, especially in those patients who already had ventricular arrhythmias prior to the LVAD implantation [31]. There is no consensus about ICD tachytherapy in MCS patients, where a shock in conscious patients is unfortunate, while on the other hand ventricular arrhythmias are detected early to prevent RHF and hypoperfusion. Most often, ICD settings are adapted to only treat very fast ventricular arrhythmias including ventricular fibrillation. Apart from ventricular arrhythmias, atrial fibrillation is also common in MCS, and depending on the clinical effect, might require rhythm control [2].

## Referral

Given the fact that the optimal timing of LVAD implantation is crucial and that the outcome after LVAD implantation in patients with rapidly progressive heart failure (INTERMACS I) is far inferior to outcome in patients with less severe heart failure, early referral to a transplant and MCS centre is mandatory. Several characteristics suggesting referral are:

- Severely symptomatic: NYHA III+ to IV despite optimal heart failure treatment;
- Relatively young patients with symptomatic heart failure;
- Genetic cardiomyopathies with a likelihood of rapid progression of disease (e.g. PLN mutation);
- Recurrent admissions for heart failure;
- Inotrope dependency;
- Difficulties in titration of heart failure medication (as a result of hypotension, renal failure, intolerance);
- The need for high-dose diuretics (arbitrary >4 mg bumetanide/>160 mg furosemide).

The mnemonic ‘I Need Help’, is a helpful tool for timely referral (Tab. 3; [32]).

**Table 3:** Patient selection for referral to advanced heart failure centre using I NEED HELP

I	Inotropics	Previous or current need for inotropics
N	NYHA III–IV/Natriuretic peptides	Persisting NYHA III–IV or increased (NT-pro)BNP
E	End-organ failure	Deteriorating kidney and/or liver function
E	Ejection fraction	Severely depressed left ventricular function (ejection fraction <20%)
D	Defibrillator shocks	Repeated ICD shocks
H	Hospitalisations	More than 1 admission for heart failure in the last 12 months
E	Edema or escalating diuretics	Persisting congestion or increasing diuretic dose
L	Low blood pressure	Consistent low systolic blood pressure (<90–100 mm Hg)
P	Prognostic medication	Inability to titrate evidence based medication (ACE inhibitor/ARB/beta blocker/MRA or ARNI)

*NYHA* New York Heart Association, (*NT-pro)BNP* (N-terminal-pro) B‑type natriuretic peptide, *ICD* implantable cardioverter defibrillator, *ACE* angiotensin-converting enzyme, *ARB* angiotensin receptor blocker, *MRA* mineralocorticoid receptor antagonist, *ARNI* angiotensin receptor neprilysin inhibitor

## Conclusions and future directions

All patients with advanced heart failure that proves refractory to optimal conventional therapy have to be considered for heart transplantation and/or long-term MCS. Early consultation and referral to a tertiary centre for evaluation of treatment options and the correct timing of advanced therapies is mandatory. In this analysis, many factors have to be weighed, including prognosis without heart transplantation/MCS, outcome after heart transplantation/MCS with regard to mortality and morbidity as well as an idea on potential improvement after heart transplantation/MCS implantation.

Currently, survival after LVAD therapy in the Netherlands approximates 83%, 76% and 70% after 1, 2 and 3 years, respectively. However, this therapy is still associated with substantial morbidity. The intensive management of LVAD patients is restricted to implanting centres, but in case of adverse events, these patients may present to other hospitals. Therefore, all cardiologists need to be aware of the management of adverse events in MCS patients [33]. Outcome after LVAD therapy can be improved by technical adjustments in the design; infectious complications surely will be diminished if there is no longer a need for a driveline to deliver energy to the pump [34]. Personalised anticoagulation may decrease bleeding problems as well as thrombosis. In this way outcome after LVAD implantation will improve even more. Therefore, it has to be expected that long-term MCS will become more and more important as a generally accepted, frequently applied therapy in advanced heart failure.
